# Initial defensive secretory compounds emitted from the live millipede and the induction of apoptotic cell death

**DOI:** 10.1038/s41598-021-87390-w

**Published:** 2021-04-15

**Authors:** Junsei Taira, Miki Tamashiro, Kaori Naka, Sahori Gakiya, Kazuyo Taira

**Affiliations:** 1 Department of Bioresources Technology, National Institute of Technology, Okinawa College, 905 Henoko, Nago-City, Okinawa 905-2192 Japan; 2 Okinawa Prefectural Enterprise Bureau, Water Quality Control Office, 1 Ishikawahigashionnazaki, Uruma-City, Okinawa 904-1108 Japan

**Keywords:** Chemical biology, Zoology

## Abstract

The initial defensive secretory compounds emitted from a live millipede have not yet been clarified. This study focused on elucidating the initial secretory compounds emitted from a live millipede. Pre-concentration of the defensive secretory volatile organic compounds (VOC) from the live Polidesmida millipedes, *Chamberlinius hualienensis* and *Oxidus gracilis*, was performed using a three-stage VOC concentration technique by an on-line GC/MS system. As a result, the monoterpenes derived from the plant metabolite; i.e., *α*-pinene, *α*-thujene, β-pinene, 3-carene, β-myrcene, β-phellandrene, γ-terpinene, *o,m,p*-cymenes, limonene and camphene were first detected as the initial secretory substances. It was elucidated that some plant monoterpenes have a repellent effect and antifungal and antibacterial actions which are used as defensive substances. In addition, this study also confirmed that these monoterpenes induced apoptotic cell death involved in the induction of the caspase 3/7 activity. The millipede feeds on fallen or withered leaves containing the monoterpenes. Thus, the millipede accumulates the plant defensive secretions in the exocrine defense glands of the body somites, which would be used as against predators.

## Introduction

Some orders of millipedes provides a pair of exocrine defense glands in the body somites and these glands discharge secretory compounds, such as cyanogenics (mandelonitrile, benzoyl cyanide), guaiachol, hydroquinones, benzoquinones and phenols which are well known as defensive substances^1–7^. These secretory compounds have been detected from the soaked millipede using an organic solvent, such as methanol and hexane. Thus, these secretary compounds are detected dependent on the polarity of the extraction solvent, resulting in the fact that phenols, quinones (benzoquinones and hydroquinones), and cyanogenics as the main chemical groups have been reported in the various species of the millipede^6^.

Frequent outbreaks by the Polidesmida millipedes, *Chamberlinius hualienensis* and *Oxidus gracilis*, have often occurred. The outbreak involved their defensive secretory compounds, such as *p*-cresol, benzaldehyde, and mandelonitrile^8,9^. However, the initial defensive secretion emitted from the live millipede has not yet been clarified from all species of the millipedes. Thus, this study focused on elucidating the initial defensive secretory volatile organic compound (VOC) emitted from live millipedes such as *O. gracilis* and *C. hualienensis* and the toxicity of the compounds find in their VOC of the millipedes.

We employed a three-stage VOC concentration technique for the efficient collection of the emitted secretory compounds from the live millipede. Pre-concentration of the initial secretion was performed using a large-volume headspace on-line GC/MS system^10,11^.

We now describe the characteristics of the initial defensive secretory compounds emitted from the live millipedes and the induction of caspase activity leads to apoptotic cell death.

## Materials and methods

### Chemicals

3-(4,5-Dimethyl-2-thiazolyl)-2,5-diphenyl-2H-tetrazolium bromide (MTT) and authentic compounds for the secretion analysis were obtained from Wako Pure Chemicals (Osaka, Japan). The caspase-3/7 assay kit was purchased from Promega (WI, USA).

### Collections of millipedes

The polydesmida millipedes of *O. gracilis* and *C. hualienensis* were collected in kitanakagusuku, Okinawa, Japan, in May 2008. Individual millipedes of *O. gracilis* (male, n = 4 and female, n = 3) and C. *hualienensis* (male, n = 2) and female, n = 7) were put together and transferred to a vial (120 ml) for collecting the initial secretion.

### Pre-concentration of volatile secretions

Pre-concentration of the VOC and subsequent constituent analysis were performed using an ENTECH 7100A (Entech Instruments, Inc., CA, USA) online system gas chromatograph (GC) (Agilent 6890 N, Agilent Technologies, CA, USA) equipped with a mass selective detector (MSD) (5975C inert XL MSD, Agilent Technologies, CA, USA). Briefly, the live millipedes without any plant residues were maintained in a vial at room temperature for 40 min and subsequently pre-concentrated by a three-stage VOC concentration technique involving three types of modules (modules 1, 2, and 3) and a trap tube as previously reported^10^. The atmosphere (100 ml) emitted from the millipedes was absorbed in module 1 using a glass bead/tenax trap at -145 °C for 1 min, then at the heating 10 °C while slowly passing nitrogen gas through module 1. The adsorbed sample was then transferred to module 2 containing a tenax trap pre-chilled at -30 °C. The target compounds were back-flushed at 150 °C for 2.5 min prior to being further focused in a capillary focusing trap in a pre-chilled (-160 °C) module 3 (cryo-focus trap). The compounds were then at the heating 110 °C for injection into the analytical column.

### GC/MS analysis

A capillary column of DB-WAX (60 m length × 0.25 mm i.d., 0.5 μm film thickness, Agilent Technologies) and DB-1(60 m length × 0.32 mm i.d., 1 μm film thickness, Agilent Technologies) were used for the GC/MS under previous analytical conditions^10^. The oven temperature was initially set at 40 °C for 5 min, and subsequently ramped to 240 °C at 3 °C/min, followed by a final 5 min hold at 240 °C. The inlet temperature was set at 200 °C and the injection split ratio was 40:1, then introduced into the individual MS detector. Mass spectrometry was carried out in the scan mode using an electron ionization voltage of 70 V and a scan range from *m/z* 29 to 300. Analysis of the VOC was performed using POWERED PRO SOFTWARE (willy 7n. L., Agilent Technology). Identification of the compounds was performed by comparison to commercially- available authentic compounds.

### Cell culture

Each cell line (American Type Culture Collection) of RAW264.7 and PC12 was cultured in DMEM medium (including 10% FBS (Wako Pure Chemicals), 100 U/mL penicillin, and 100 ng/mL streptomycin) at 37 °C in a 5% CO_2_ atmosphere.

### Cytotoxicity of initial secretory compounds

The MTT assay was used to determine the cell viability^12,13^. Briefly, cells were seeded at a density of 5 × 10^5^ cells/ml on 96-well plates. After culturing for 24 h, the test compounds (dimethyl sulfide, 2-ethyl furan, *α*-pinene, dimethyl disulfide, β-pinene, 3-carene, β-phellandrene, γ-terpinene, camphene, 2-methyl 3-buten-2-ol, methyl thiocyanate, benzaldehyde) were dissolved in ethanol and each final concentration of sample (50, 100 and 200 μg/mL) treated for 17 h. MTT (0.5%) was added after incubation to each well, the samples were incubated for 2 h, then the cell suspensions were carefully removed. The extraction with DMSO (100 μl) was subsequently measured at 570 nm with the reference at 630 nm using a microplate reader (BIO-RAD MODEL 680XR, BIO-RAD, CA, USA). Cell survival expressed as % of the control cell for solvent treated cells instead of test compound.

### Caspase activity

Cells were plated at a density of 5 × 10^5^ cells/ml on 96-well plates after culturing for 24 h, the test samples were prepared as with the MTT assay and they were treated for 17 h. The caspase activity in the cultured cells was measured using the commercially- available caspase-3/7 assay kit^12,13^. Cells were incubated with 100 μl of proluminescent substrate containing Z-DEVD provided in the kit. Following the caspase cleavage, the luminescence of the reaction products was measured using a microplate reader (GLOMAX MULTI DETECTION SYSTEM, Promega, WI, USA). Caspase activity expressed as % of the control for solvent treated cells instead of test compound.

### Statistical analysis

The data are expressed as mean ± SD of four samples. Significant differences between the control and test samples were analyzed by the Student’s *t*-test with the significance at *p < 0.05 and **p < 0.01.

## Results

**Figure 1:**
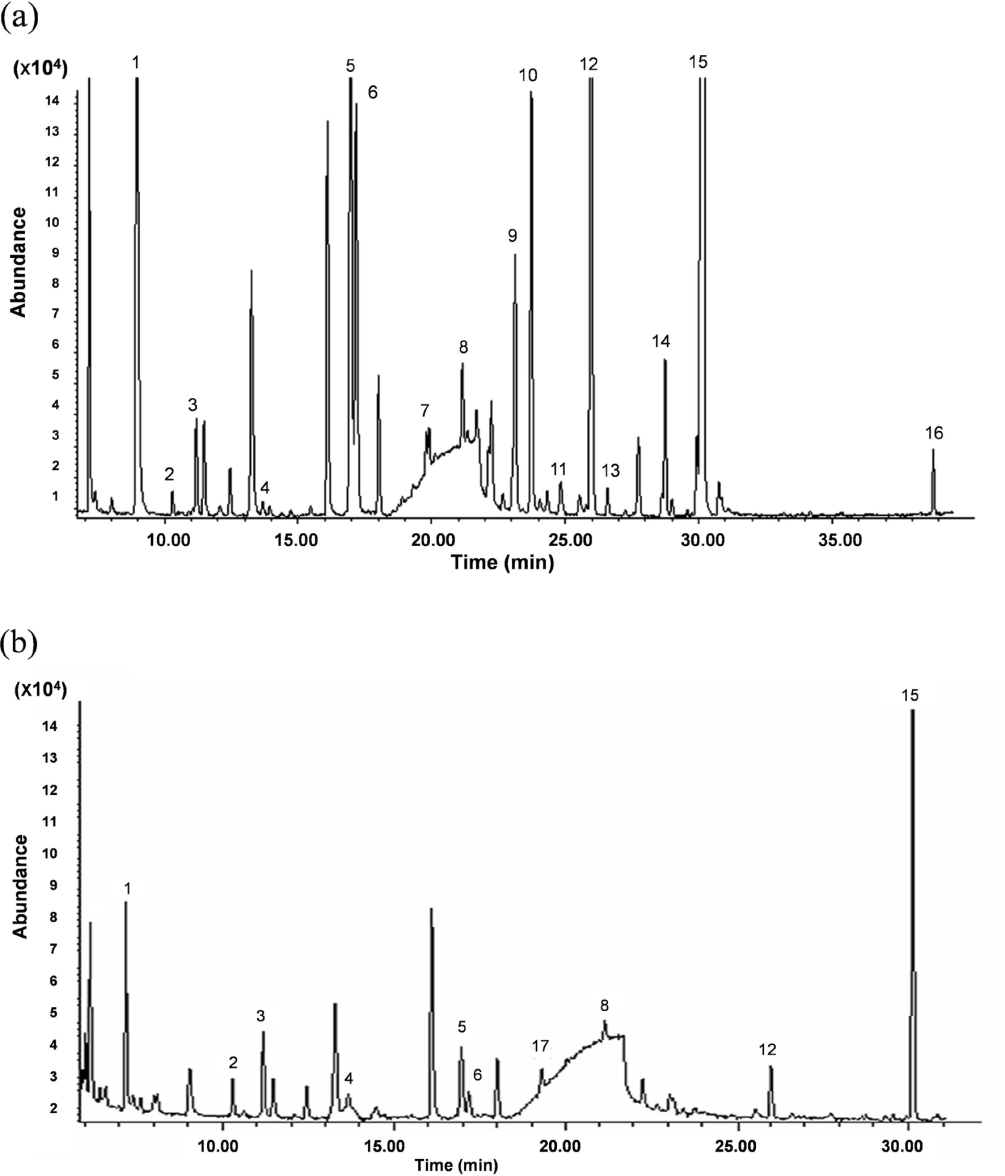
(**a**) Total ion chromatogram (TIC) of the initial defensive secretory compounds emitted from the live millipede, *Oxidus gracilis.* The defensive secretory compounds were analyzed by a large-volume headspace on-line GC/MS system. The analysis was performed by comparison to commercially available authentic compounds and a library research of the compounds with asterisk (*). Individual peaks of the initial secretory compounds are as follows: 1, dimethyl sulfide; 2, 2-methyl furan; 3, 3-methyl furan; 4, 2-ethyl furan; 5, *a*-pinene; 6, *a*-thujene; 7, dimethyl disulfide; 8, β-pinene; 9, 3-carene; 10, *β-myrcene; 11, *m*-cymene; 12, limonene; 13, *β-phellandrene; 14, γ-terpinene; 15, *o-cymene and 16, *p-cymene. (**b**) TIC of the initial defensive secretory compounds emitted from the live millipede, *Chamberlinius hualienensis*. 1, dimethyl sulfide; 2, 2-methyl furan; 3, 3-methyl furan; 4, 2-ethyl furan; 5, *a*-pinene; 6, *a*-thujene; 8, β-pinene; 12, limonene and 17, camphene.

**Figure 2:**
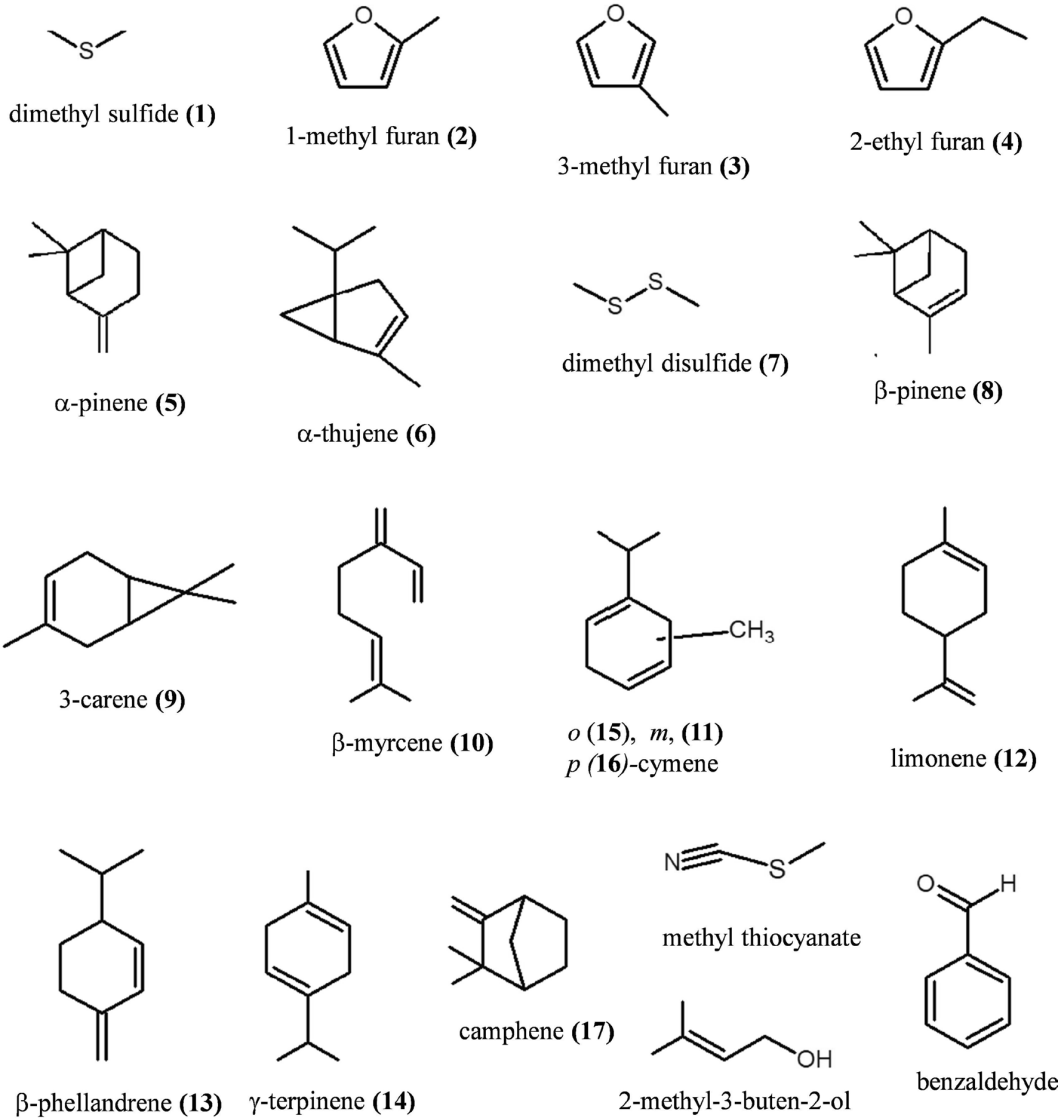
The chemical structures of the initial secretory compounds emitted from the live millipedes, *Oxidus gracilis* and *Chamberlinius hualienensis*. Each number of the compounds corresponds the peaks of TIC in Fig. 1a,b.

The initial defensive secretory compounds emitted from the live millipedes were detected by the pre-concentration of the secretion using a large volume headspace on-line GC/MS system. The total ion chromatogram (TIC) of the initial secretory compounds emitted from both live millipedes of *O. gracilis* and *C. hualienensis* are indicated in Fig. 1a,b, respectively. The identity of the initial secretory compounds from the millipede was carried out by their similarity to commercially available authentic compounds and a library search, resulting in the determination of 11 monoterpenes, i.e., *α*-pinene; *α*-thujene; β-pinene; 3-carene; β-myrcene; *m*-cymene; limonene; β-phellandrene; γ-terpinene; *o*,*m*,*p*-cumenes; camphene including 2 sulfur compounds of dimethyl sulfide and dimethyl disulfide, and 3 furan compounds of 2-methyl furan, 3-methyl furan and ethyl furan. In addition, benzaldehyde, 2-methyl-3-buten-2-ol and methyl thiocyanate were detected from both millipedes using the non-polar column DB-1 (data not shown). The chemical structures of the initial secretory compounds detected in this study depicted in Fig. 2. These initial secretary compounds were different from the main secretory compounds, such as phenols and quinones (benzoquinones and hydroquinones) detected from the soaked millipede using an organic solvent^2^.

**Figure 3:**
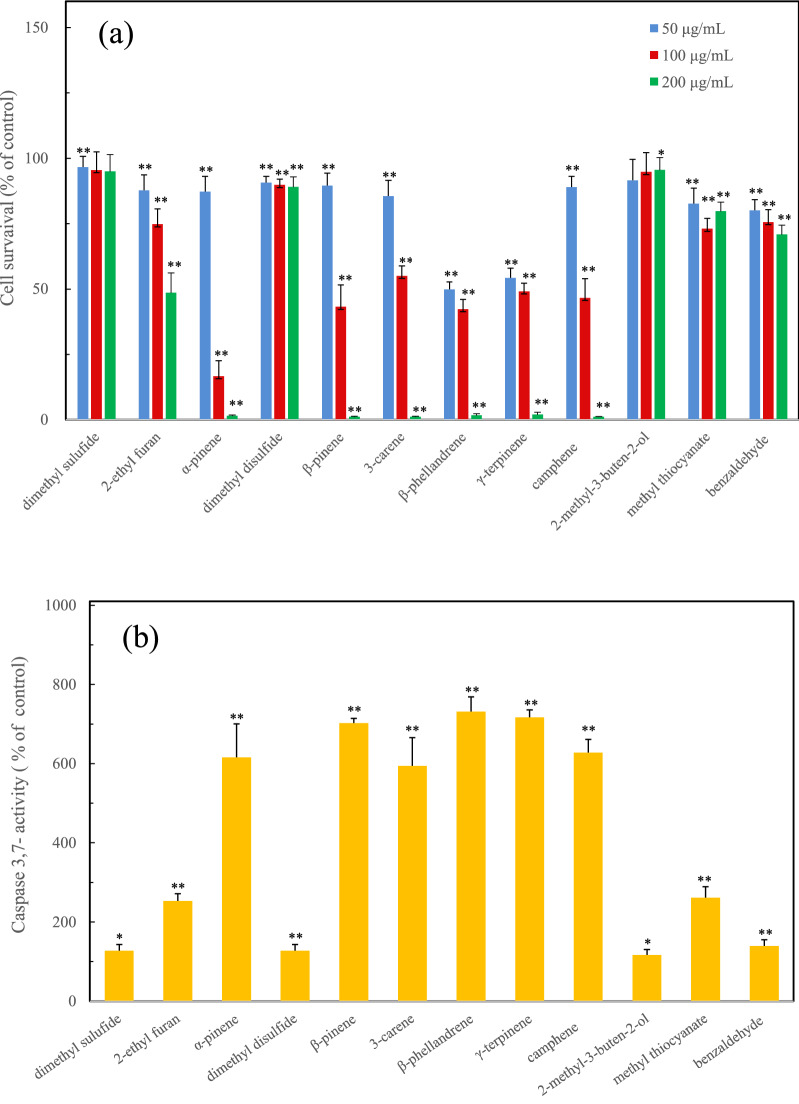
The induction of cytotoxicity with apoptotic cell death due to the initial defensive compounds from the live millipede against RAW 264.7 cells, (**a**) cytotoxicity of the initial defensive compounds. (**b**) caspase activity of the initial defensive compounds. Significant difference was determined by *t*-test. *p < 0.05 and **p < 0.01 for the control cell.

To explore the other biological activities of the initial defensive secretory compounds, their cytotoxicity was examined using two cell lines of a mouse macrophage-like cell, RAW264.7 and PC12 cells, derived from a pheochromocytoma of the rat adrenal medulla. As a result of the evaluation for the RAW264.7 cells, the monoterpenes of *α*-pinene, β-pinene, 3-carene, β-phellandrene, γ-terpinene, camphene indicated a high cytotoxicity in a dose dependent manner. These cytotoxic secretory compounds also induced the caspase3/7 activity related to the apoptotic cell death (Fig. 3).

**Figure 4:**
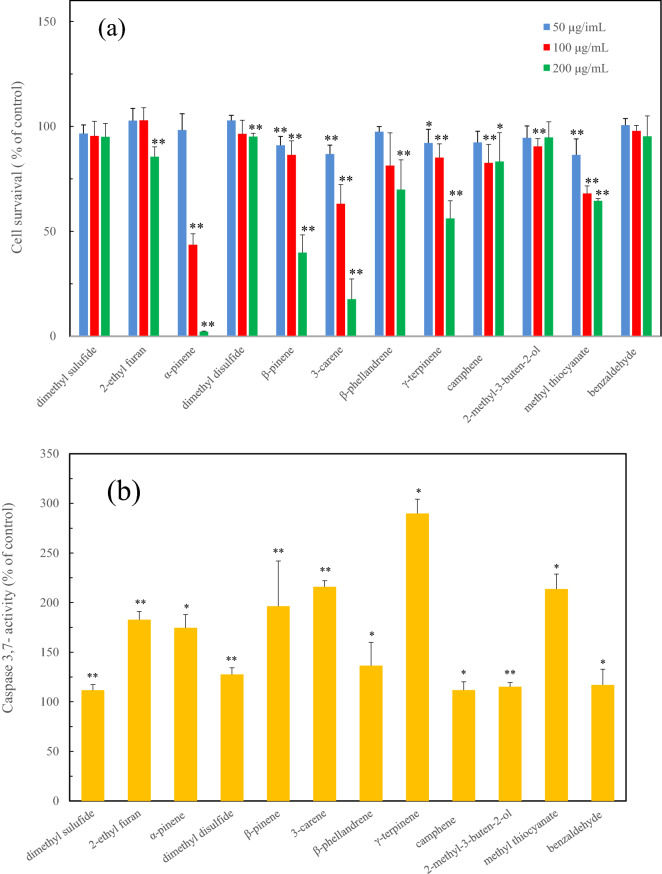
The induction of cytotoxicity with apoptotic cell death due to the initial defensive compounds from the live millipede against PC12 cells, (**a**) cytotoxicity of the initial defensive compounds. (**b**) caspase activity of the initial defensive compounds. Significant difference was determined by *t*-test. *p < 0.05 and **p < 0.01 for the control cell.

In addition, the cytotoxicity of the initial secretory compounds was also confirmed using the PC12 cells which are widely utilized for studies of different aspects of neuronal physiology (Fig. 4). The cytotoxicity of the monoterpenes of *α*-pinene, β-pinene, 3-carene and γ-terpinene indicated a high cytotoxicity involved in the apoptotic cell death with the caspase activation. This result was similar to the result of the RAW264.7 cell treated with the test compound (Fig. 4).

## Discussion

In this study first demonstrated the initial secretory substance from the live millipedes Polidesmida millipedes, *Chamberlinius hualienensis* and *Oxidus gracilis* as depicted in Fig. 2. These initial secretory compounds, such as monoterpenes, sulfur compounds, and furan compounds were first detected in all species of the millipede^2^. In our previous studies, the secretions of phenol, cresol, benzaldehyde and dimethyl acetal in the methanol-soaked millipedes of both *C. hualienensis* and *O. gracilis* have been identified as defensive secretory compounds^7,8^. The secretory compounds detected in the soaked millipede were different from those of the initial secretory compounds emitted from the live millipede. In addition, both millipedes collected the following year at the same place were analyzed and new initial secretory compounds of 3-metyhl-2-butaneone, 2-pentanone, 2-butanone, methanethiol, methoxy benzene, and involving methyl thiocyanate from *O*. *gracilis* were detected (data not shown). In our previous studies, the concentrations of the internal elements in the millipede and the soil including leaf litter in the habitat were evaluated. The soil concentration in the habitat reflected the characteristics of the internal elements and metal accumulation in both millipedes^14,15^. Therefore, both millipedes used in this study, which were collected in the same habitat environment, may contain the similar initial secretary compounds.

The cytotoxicity of the initial defensive secretory compounds was examined using two beneficial cell lines of RAW264.7 macrophages for cell death through the inflammatory response and PC12 cells as index of neuronal toxicity. In previous studies have shown in vitro and in vivo antitumor activity of monoterpenes in essential oils obtained from plants^16^. In this study, the monoterpenes of *α*-pinene, β-pinene and γ-terpinene indicated a high cytotoxicity in the apoptotic cell death which were also reported antitumor activity, such as leukemia, melanoma and colon cancer^16^. The monoterpenes are widely contained in various plants, and some monoterpenes have a repellent effect and antifungal and antibacterial action^17–19^. The millipede feeds on fallen or withered leaves containing the monoterpenes. Thus, the millipede accumulates the plant defensive secretions in the exocrine defense glands of the body somites. The initial secretory compounds emitted from millipede would be used as initial defensive substances against predators as well as the chemical defense of plants. In addition, it is well known that millipedes discharge defensive odorous secretions during outbreak that cause headaches and unpleasant feelings for humans^7–9^. Therefore, the biological activity of the initial secretions may be responsible for causing unpleasant sensations.

In conclusion, this study first elucidated the initial defensive secretory compounds emitted from the live millipedes. In particular, the monoterpenes induced apoptotic cell death. The millipede accumulates these cytotoxic monoterpenes derived from plant secondary metabolites and they would be used as defensive substances against predators as well as the chemical defense of plants.
